# Health among workers retiring after the state pension age: a longitudinal and comparative study

**DOI:** 10.1186/s12877-022-03690-4

**Published:** 2022-12-20

**Authors:** Isabel Baumann, Ariane Froidevaux, Ignacio Cabib

**Affiliations:** 1 Institute of Public Health, School of Health Sciences, Zurich University of Applied Sciences; National Centre of Competence in Research “Overcoming Vulnerability: Life Course Perspectives”, Gertrudstrasse 15, 8400 Winterthur, Switzerland; 2 Centre for the Interdisciplinary Study of Gerontology and Vulnerability, University of Geneva, Katharina-Sulzer-Platz 9, 8400 Winterthur, Switzerland; 3 Department of Management, College of Business, University of Texas at Arlington, 701 S W St Arlington, Arlington, TX 76010 USA; 4 Instituto de Sociología & Departamento de Salud Pública, Pontificia Universidad Católica de Chile, Santiago, Chile; 5 Centro UC Estudios de Vejez y Envejecimiento, Pontificia Universidad Católica de Chile, Santiago, Chile

**Keywords:** Health status, Employment, Retirement, Welfare regimes, Old-age pension redistribution, Sequence analysis

## Abstract

**Background:**

In recent decades, many countries have observed increasing labor force participation beyond the state pension age (SPA). However, there is a lack of research on employment beyond SPA and how it relates to older workers’ health. Moreover, there is a need to better understand how institutional factors affect the relationship between older workers’ employment and health. In this study, we examine simultaneous employment and health trajectories over 11 years in 12 countries from Europe and the Americas, and study how these trajectories differ by welfare state regime and level of old-age pension redistribution.

**Methods:**

We used a harmonized pooled-country dataset of 3699 older workers based on four representative panel surveys: the Survey of Health, Ageing and Retirement in Europe (SHARE), the English Longitudinal Study of Ageing (ELSA), the Health and Retirement Survey (HRS), and the Chilean Social Protection Survey (EPS). We conducted multichannel sequence analysis to estimate the types of simultaneous employment–health trajectories, and multinomial regression analysis to examine the relationship between trajectory types and institutional factors.

**Results:**

We found that late retirement was equally associated with poor and good health. There is also a higher prevalence of late retirement trajectories in combination with poor health in liberal welfare regimes and in countries with lower levels of old-age pension redistribution.

**Conclusion:**

Our study indicates that nonliberal welfare regimes and redistributive old-age pension policies may be better suited to protect vulnerable workers while providing those in good health with the opportunity to work beyond the SPA.

**Supplementary Information:**

The online version contains supplementary material available at 10.1186/s12877-022-03690-4.

## Background

In line with the general improvement in older adults’ health and the rise in life expectancy, the last two decades have seen increasing labor force participation beyond the state pension age (SPA) in many developed economies [1]. SPA describes the age at which people are entitled to receive full old-age pension benefits, which does not necessarily imply that retirement at this age is mandatory [2]. To address the increasing financial pressure on old-age pension systems that emerging since the 1990s, some countries (e.g., Sweden or Denmark) have introduced retirement policy reforms, including measures to incite employment beyond SPA, and thus reversing the trend of early retirement observed between the 1970s and 1990s [3–5]. Interestingly, the trend of prolonging working lives can also be observed in countries that have not recently reformed retirement policies, such as Switzerland or the United States [1].

### Health and employment in later life

Health status has been identified as one of the factors with the strongest impact on employment and retirement decisions among older workers [6, 7]. For instance, it has been shown that adverse health conditions often go along with early retirement [8–10], while late retirement and bridge employment (i.e., taking up of a new job while receiving a retirement pension) are positively associated with health [11, 12]. Nevertheless, a change in health status does not always lead to a change in employment status [13]. Research has shown that the extent to which the change in health status affects a change in employment status depends on factors such as the nature of the health problem [14], workers’ occupation [15], or country-level institutional factors [16]. For instance, a study from Finland shows that sickness absence from work in the case of musculoskeletal diseases differs strongly between occupations [14]. While musculoskeletal diseases only led to a low share of sickness absences among upper non-manual workers, it related to about three times as many absences among manual workers.

However, despite several advances on the understanding of the role of health conditions on working pathways in later life, most research in this field has measured health status cross-sectionally or at a single point in time—typically at the beginning or end of the observation period—to then explore how health status either affects the unfolding of prospective employment trajectories, or is affected by employment trajectories [3, 11, 12, 17]. Such conceptualizations are problematic given that both employment and health are dynamic or time-variant factors that mutually affect each other [18] and thus both need to be examined longitudinally [19]. Two pieces of research that examined retirement transitions and health both longitudinally, are on the one hand a study based on data from the United States and on the other hand a study based on data from Sweden [20, 21]. Both studies use sequence analysis to examine the retirement transitions. Although health was measured longitudinally, these two studies report levels of health within different types of retirement transitions as a population average without displaying intraindividual variance.

Accordingly, there is a need to better understand how changes in individuals’ employment status may be intertwined with changes in their health status in later life. To do so, we adopt a life course perspective and model simultaneous employment–health trajectories by using multichannel sequence analysis, including individuals who died during the observation period, and following them up to 11 years after SPA (i.e., up to age 76 for individuals with a SPA of 65). The life course perspective is particularly helpful, as it conceptualizes retirement as one transition reflecting the many age-related changes occurring from birth to death [22], and focuses on trajectories with their positive and negative changes [10], within life domains such as work and health [6]. Including potential deaths during the examined period does not only allow to capture a dimension of health that is not assessed by conventional health measures (e.g., self-rated health) but also addresses sample selection bias due to mortality [23]. Furthermore, most past studies have focused on a shorter timeframe, as well as on younger age groups, so that older adults were followed up to a maximum age of 70. The increasing labor force participation of workers beyond the age of 70 calls for an inclusion of longer timeframes [16, 24].

### Institutional factors

Another important characteristic of the life-course perspective is its emphasis on individuals’ agency being limited by the social context in which intertwined retirement sequences occur [25]. Hence, the association between employment status and health is likely to depend on the broader institutional context in which individuals are embedded [10]. However, there is currently a lack of research examining the influence of the socioeconomic context (beyond socioeconomic status such as education) on retirement [26, 27] with few exceptions examining the role of exposure to economic recessions [11, 28]. In this research, we explore the influence of two distinct institutional factors on employment–health trajectories in later life: welfare regimes and old-age pension redistribution. By doing so, we address an important limitation of prior work, which has solely considered welfare regimes, hence overlooking old-age pension redistribution as another key institutional factor possibly affecting individuals’ retirement. To our knowledge, this is the first study exploring the role of old-age pension redistribution in late-life trajectories.

#### Welfare regime

Crucial welfare state dimensions, which strongly vary across welfare regimes, such as the level of decommodification (i.e., the extent to which individuals rely on paid work to obtain a decent standard of living or the share of private vs. public health care spending) or the requirements to access public benefits (whether universal, unconditional, or targeted access), are factors that potentially drive the co-development of employment and health in later life. We specifically rely on a five-group classification of welfare regimes consisting of liberal (i.e., providing only a basic income, with comparatively little redistribution across income levels; e.g., USA), corporatist (i.e., providing more generous welfare benefits but only to those who have followed a standard occupational career; e.g., France), social-democratic (i.e., comparatively generous social security scheme, universal access to welfare; e.g., Sweden), liberal-corporatist (i.e., combining elements of liberal and corporatist welfare regimes; e.g., Switzerland), and Southern European (i.e., support to those who do not have a paid employment is often provided by the family rather than the state, e.g., Spain) countries [29–33].

While the influence of different welfare regimes on working-age population health has been thoroughly examined during the last two decades (see, for instance, [34, 35]), only very recently have researchers started to explore how welfare regimes may shape later-life trajectories, reporting preliminary evidence that welfare regime orientation may play an important role in the association between employment trajectories and health status among older people [3, 7, 16, 36–38]. One of these studies showed that in social-democratic (and corporatist) welfare regimes, early retirement was associated with good health, while it was associated with poor health in liberal and liberal-corporatist countries [36]. Overall, past research suggests that, in welfare regimes with more generous welfare benefits, older workers are likelier to maintain good health [39], which is probably due to less pressure experienced by older workers to remain in the labor force.

However, several limitations of these previous studies need to be addressed. First, Esping-Andersen’s typology was developed in the 1990s, which was earlier than most countries, such as Sweden (mid 1990s), started to reform their old-age pension systems [40, 41]. Thus, the typology may lack accuracy in explaining current retirement behavior. Second, while welfare regime theory considers redistribution between socioeconomic groups within a society, the measure of redistribution is rough. Third, these studies focused on the influence of the welfare state as a whole without examining the contribution of specific policies, such as old-age pension policies or policies to reconcile paid employment and care work. A recent study has taken this problem into account by examining how the level of social expenditure in different fields affects older people’s health [32]. However, it did not find a significant relationship between the overall level or the old-age pension-specific level of social expenditure on the one hand, and self-rated health or grip strength on the other hand. One reason for the lack of association may be that these measures of social expenditure do not capture variations in social expenditure across different socioeconomic groups within a society. While the concept of a welfare regime may be useful for capturing the general socioeconomic context, there is a need for additional knowledge of the more detailed mechanisms that may explain individuals’ trajectories. In this study, we therefore use a measure that captures the redistributive effect of old-age pension benefits: the level of old-age pension redistribution.

#### Old-age pension redistribution

Old-age pension redistribution refers to the extent to which the old-age pension redistribution system is fair in terms of more or less inequalities between higher and lower pre-retirement earnings [42]. Concretely, it is measured as the ratio between the net replacement rates (NRR) at different pre-retirement earnings levels. To understand this ratio, first, the NRR represents individuals’ net pensions as a share of their net earnings when they were employed, corrected for income taxes, and social security contributions [42]. Concretely, an NRR of 100% means that people receive an old-age pension of the same amount as their pre-retirement earnings. We draw on the NRR calculated by the OECD [42]. In the countries included in our study, the NRR varied between 51% (U.S.) and 93.2% (Austria) for the average pre-retirement earnings (see Table 1). NRR have been used in previous research such as in a European study on retirement decisions among couples [43].

**Table 1:** Overview of the selected countries

Country	Welfare regime	State pension age (SPA)		Net replacement rates (NRR) in % ^**f**^			Old-age pension redistribution ratio ^**g**^
		*Women*	*Men*	*0.5 times average of pre-retirement earnings*	*Average of pre-retirement earnings*	*1.5 times average of pre-retirement earnings*	
United States	Liberal	Between 65 and 66 ^**a**^	Between 65 and 66 ^**a**^	61.4	51.0	44.9	1.4
Chile	Liberal	60	65	74.4	64.3	62.7	1.2
Austria	Corporatist	60	65	91.2	93.2	93.5	1.0
Belgium	Corporatist	63	65	82.7	63.1	53.3	1.6
France	Corporatist	60^**b**^	60^**b**^	98.0	68.8	62.6	1.6
Germany	Corporatist	65^**c**^	65^**c**^	61.7	71.8	79.2	0.8
England	Liberal-corporatist	60	65	78.4	47.6	38.2	2.1
Switzerland	Liberal-corporatist	63	65	71.4	67.3	53.0	1.3
Denmark	Social-democratic	65^**d**^	65^**d**^	95.6	58.2	42.5	2.2
Sweden	Social-democratic	65^**e**^	65^**e**^	90.2	68.2	70.1	1.3
Italy	Southern European	65	65	89.3	88.8	88.4	1.0
Spain	Southern European	65	65	88.7	88.3	88.4	1.0

Note: SPA is the age at which full pension benefits can be claimed. We indicate SPA for 2004 [42]: ^a^ Early pension from age 62 with reduced benefits. ^b^ A full state old-age pension requires 37.5 years of contributions. ^c^ Applies for individuals with five years of contributions; 63 for individuals with 35 years of contributions. ^d^ 67 for people born before July 1, 1939; a full state old-age pension requires 40 years of residence. ^e^ Applies for basic pensions; an earnings-related public pension can be claimed from 61. ^f^ NRR are individuals’ net old-age pensions as a share of their net earnings when they were employment, corrected for income taxes and social security contributions. We indicate the numbers for men [42] (for Chile, we use data from 2010 since no earlier data is available [42]. ^g^ Old-age pension redistribution is measured as the ratio of NRR of 0.5 times the average pre-retirement earning divided by NRR of 1.5 times the average pre-retirement earning. The higher the number, the more redistributive the retirement policy. Reading example: In Germany, the NRR of a person with 0.5 times the average pre-retirement earning is 61.7% and the NRR of a person with 1.5 times the average pre-retirement earning is 79.2%. This implies that among people living in German, those with lower pre-retirement earnings receive relatively less old-age pension than those with higher pre-retirement earnings. Germany has the lowest old age pension redistribution (0.8) of all countries examined in the present study, meaning that Germany has lower redistribution from individuals with high pre-retirement earnings to those with low pre-retirement earnings than other countries (e.g., Denmark, with an NRR of 2.2)

Second, the concept of old-age pension redistribution itself, which we introduce here as a novel contribution to the literature, represents the ratio between different levels of NRR, which will vary by country. The higher the number, the more redistributive the retirement policy—that is, the fairer the pension redistribution system (i.e., fewer inequalities between higher and lower pre-retirement earnings). Hence, to obtain the old-age pension redistribution ratio, we used the NRRs provided by the OECD for each country for different levels of pre-retirement earnings [42]. To be specific, the ratio is calculated between the NRR for workers with 0.5 times the average pre-retirement earnings to represent lower income levels (e.g., 91.2% for Austria or 61.4% for the U.S.), and the NRR for 1.5 times the average pre-retirement earnings to represent higher income levels (e.g., 93.5% for Austria or 44.9% for the U.S.) and define old-age pension redistribution as the ratio between these two levels (for a detailed example, see the methods section). As shown in Table 1, Austria thus has an old-age pension redistribution ratio of 1.0 (i.e., comparably lower), while the U.S. has a ratio of 1.4 (i.e., comparably higher), meaning that in the U.S., there is more redistribution from individuals with high pre-retirement income to those with low pre-retirement income than in Austria. Put differently, Austria provides proportionally more pension for retirees with higher income before retirement than the U.S. do.

Finally, welfare regimes and the old-age pension redistribution ratios are distinct institutional factors, so they do not necessarily overlap [30, 44, 45]. For instance, *social-democratic* Sweden has a comparably low old-age pension redistribution ratio of 1.3 (i.e., a less fair redistribution system with more inequalities between higher and lower pre-retirement earnings), while *social-democratic* Denmark has a comparably high old-age pension redistribution ratio of 2.2.

### Aims and hypotheses

Adopting a life course perspective, our first aim is to map employment and health trajectories, with a focus on employment beyond the SPA. To do so, we examine individuals’ trajectories in the years after SPA in 12 industrialized countries from Europe and the Americas, based on a harmonized pooled-country dataset. We followed older individuals’ employment and health trajectories from 2004 to 2015, using a sequence-and Cluster-analytic approach. Our second aim is to identify how institutional factors (welfare regimes and old-age pension redistribution) may be associated with simultaneous health–employment trajectories.

To do so, we developed the following two hypotheses: First (H1), with respect to the employment–health trajectories, based on the above-discussed literature, we hypothesize that, overall, late retirement in good health is more frequent than late retirement in poor health. Second (H2), with respect to the institutional factors associated with late employment–health trajectories, in line with welfare regime theory according to which individuals in social-democratic health regimes show the best health and lowest health inequalities due to greater financial protection [39], we hypothesize that late retirement in poor health is more frequent in countries with (H2a) liberal welfare regimes than in any other age regime, and (H2b) comparatively lower old-age pension redistribution ratios because countries with these characteristics may force older workers to remain in the labor force until later ages to make their living, independently of their health status, without providing financial protection.

## Methods

### Countries and datasets

A total of 12 countries from Europe and the Americas were included in the present study. The countries of each type of the five welfare regimes were selected. Secondary data for the 12 selected countries were taken from four representative panel surveys, mostly focusing on aging, and pooled into one dataset containing harmonized variables. Our dataset consists of a panel-balanced sample covering the period from 2004 to 2014/2015. An overview of the four panel surveys and waves is provided in Table A.1.

The inclusion of participants in the sample was based on two criteria. First, participants had to be alive and must have reached the SPA (see Table 1) in the baseline year of 2004 (i.e., at the first point of observation examined in our study), so that they did not all have the same age as they entered our study. The number of individuals fitting this first selection criterion was 5833. Second, following a conservative strategy, participants had to have at most one missing value in either their health or employment statuses across the examined survey waves. The number of individuals fitting the first and second selection criteria was 3827.

To replace missing observations in the variables we used to create the employment and health trajectories (i.e., item non-response), we performed 50 iterative multivariate imputations by chained equation models, specifically with the predictive mean matching method, and considered gender, education, survey wave, and, if applicable, health, and employment statuses as predictors before and after the missing observation. We applied imputations only if there was maximum of one missing value across the six observations of employment and health status. Finally, missing values in the explanatory variables included in our models of analysis left us with a total of 3699 participants, thus representing our final sample.

The Survey of Health, Ageing and Retirement in Europe (SHARE) provided data across five waves for 849 participants from Europe. The English Longitudinal Study of Ageing (ELSA) provided data across six waves for 579 participants from England. The Health and Retirement Survey (HRS) provided data across six waves for 1847 participants from the USA. The *Encuesta de Protección Social* (EPS) [Social Protection Survey] provided data across four waves for 424 participants from Chile. Therefore, our findings can be generalized to a cohort of individuals who reached the SPA in 2004. Due to the variation in the construction of weights across panel surveys used in this research and their generally high response rates, it is appropriate to use unweighted data [46].

### Measures

#### Employment and health trajectories

To construct simultaneous later-life trajectories, we used two variables: employment status and health status. We relied on five mutually exclusive employment statuses based on self-reported information from the survey respondents to construct the employment trajectories over the 11-year timeframe. In the survey questionnaire, respondents were able to choose from the following options: (a) working, for individuals in a full-time or part-time job who were not retired yet; (b) partly retired, indicating people who received pension benefits and continued working at least partially in the labor force (e.g., bridge employment); (c) retired, indicating people fully retired from the labor force; (d) out of the labor force, indicating unemployed, disabled, and inactive people (but not retired); (e) deceased, indicated participants who passed away at some point before a specific point in the study.

Four self-reported mutually exclusive health statuses were used to create health trajectories over the 11-year timeframe: (a) excellent or very good health, (b) good health, (c) fair or poor health, and (d) deceased. Since (b) good health is the intermediate category used in the survey questionnaires, we considered it to be a neutral health status.

#### Explanatory variables

Based on Esping-Andersen’s extended typology and previous literature, the USA and Chile were coded as liberal welfare regimes [29, 47]. Austria, Belgium, France, and Germany were coded as corporatist welfare regimes [31]. England and Switzerland were coded as liberal-corporatist countries [20, 33]. Denmark and Sweden were coded as social-democratic welfare regimes [47]. Spain and Italy were coded as Southern European welfare regimes [24].

The NRR represents individuals’ net pensions as a share of their net earnings when they were employed, corrected for income taxes, and social security contributions [32]. A NRR of 100% means that people receive an old-age pension of the same amount as their pre-retirement earnings. NRR are calculated by the OECD for different levels of pre-retirement earnings. Our explanatory variable, old-age pension redistribution ratio, is measured as the ratio of the NRR for 0.5 of the average pre-retirement earning divided by the NRR for 1.5 of the average earning. The higher the number, the more redistributive the retirement policy is. For instance, Austria’s redistribution of 1 (see Table 1) was calculated as follows: the NRR for 0.5 of the average earnings of 91.2% was divided by the NRR for 1.5 of the average earnings of 93.5%, which equals 0.97, which was then rounded to 1. Both measures (91.2 and 93.5%) are provided by the OECD. To be specific, all indicators are based on data for the year 2004 [42] – the baseline year in our analysis, except the NRR of Chile, where data was available only for the year 2010 [42].

#### Control variables

We controlled for sex, age, education (coded as: primary, secondary, and tertiary), household income (measured in quintiles within each country to understand the relative rather than the absolute economic status), marital status (coded as: married/partnered, divorced/separated, never married, and widowed), and number of chronic diseases at baseline (measured on a scale from 0 to 7 diseases, including high blood pressure or hypertension, diabetes, or high blood sugar, cancer or a malignant tumor, stroke or transient ischemic attack, chronic lung disease, and arthritis or rheumatism). All control variables were measured in the baseline year (i.e., 2004). Table A.2 in the Appendix presents the distribution of the control variables in the eight Clusters of interlocked employment and health trajectories.

### Statistical analysis

In the first step, we used multichannel sequence analysis (MCSA) to estimate Clusters of simultaneous employment–health trajectories (Gauthier et al., 2010). See the Appendices for an in-depth explanation of MCSA. In the second step, we explored the proportions of the Clusters to test statistical differences between Cluster 5 and 7. Third, we examined the relationships between our explanatory variables (welfare regime and old-age pension redistribution) and the Clusters of simultaneous employment–health trajectories, with a focus on late retirement trajectories, using multinomial logistic regressions analyses. Given that our study explores health among workers retiring after the SPA, we present only the results of the “late retirement trajectories” (i.e., trajectories of retirement after the SPA) (full results are available from the first author upon request). To facilitate the interpretation of the regression results, we calculated the predicted probabilities, which can be interpreted as changes in percentage points [48].

## Results

### Descriptive statistics

In the USA, Cluster 4 was most frequent (19.8%), in Chile Cluster 7 (49.5%), in Austria Cluster 1 (32.7%), in Belgium Cluster 2 (38.4%), in France Cluster 2 (37.4%), in Germany Cluster 2 and 3 (each 25.6%), in England Cluster 1 (32%), in Switzerland Cluster 1 (44.4%), in Denmark Cluster 1 (50%), in Sweden Cluster 1 (49.3%), in Italy Cluster 2 (33.3%), in Spain Cluster 8 (38.2%). Among people with primary education, Cluster 4 was most frequent (22.1%) and among people with secondary (21.4%) and tertiary education (33.1%) Cluster 1. Among women Cluster 1 was most frequent (20.4%) and among men Cluster 4 (22.3%). Mean age at baseline was highest in Cluster 4 (64.6 years). Cluster 1 was most frequent among married/partnered (21.1%) and never married people (18.2%), Cluster 4 was most frequent among divorced/separated (23.8%) and widowed people (19.7%). The mean number of chronic diseases at baseline was highest in Cluster 4 (2.1). Table A.2 provides an overview of the descriptive statistics.

### Health and employment trajectories

Figure A.1 in the Appendix shows the results of the selection criteria used to decide on the optimal solution of Clusters of employment–health trajectories, which led us to choose the solution with eight Clusters.

**Fig. 1:**
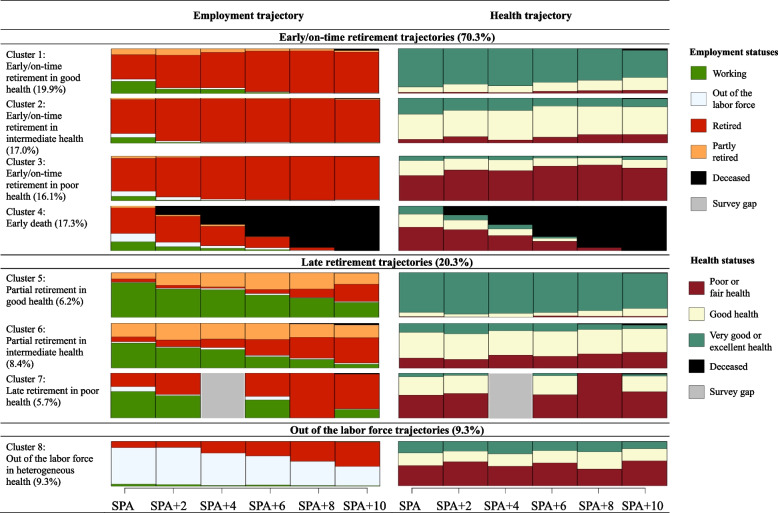
Clusters of simultaneous employment–health trajectories. ‘Survey gaps’ occur when individuals classified in a trajectory type come from a survey with no waves in a specific year

Figure. 1 presents the eight Clusters of simultaneous employment–health trajectories in chronogram plots, which are aggregated by statuses (see Fig. A.2 in the Appendix for sequence index plots, non-aggregated by statuses).

As can be observed in Fig. 1, the first four Clusters included individuals who had retired early or on time and were associated with various health states (70.3%, *n* = 2603). Specifically, the first Cluster (19.9%, *n* = 738) was called *early/on-time retirement in good health*. The second Cluster (17.0%, *n* = 628) was called *early/on-time retirement in intermediate health.* The third Cluster (16.1%, *n* = 597) is called *early/on-time retirement in poor health*. The fourth Cluster (17.3%, *n* = 640) was called *early death*.

The next three Clusters included individuals who retired later and were associated with various health statuses (20.3%, *n* = 753). Specifically, the fifth Cluster is called *partial retirement in good health* (6.2%, *n* = 230)*.* The sixth Cluster is called *partial retirement in intermediate health* (8.4%, *n* = 312). The seventh Cluster is called *late retirement in poor health* (5.7%, *n* = 211). Finally, the eighth Cluster (9.3%, *n* = 345) was called *out of the labor force in heterogeneous health*.

Overall, late retirement in good health (Clusters 5, 6.2%; 95% CI [0.055, 0.070]) was similarly frequent as late retirement in poor health (Cluster 7, 5.7%; 95% CI [0.050, 0.065]), thus rejecting our first hypothesis (H1). Furthermore, there exists substantial variability in late retirees’ health, with another share of a similar size (8.4%) in intermediate health.

### Late retirement trajectories by independent measures multinomial logistic analysis

Given that our H2 focused solely on *late* retirement, we next examined all combinations of *late* retirement with health (i.e., Clusters 5, 6, and 7). While our hypothesis referred to workers in poor health trajectories (Cluster 7), our findings also showed interesting contrasts with the other two late retirement trajectories in good and intermediate health (Clusters 5 and 6, respectively). As such, below, we present both results.

**Fig. 2:**
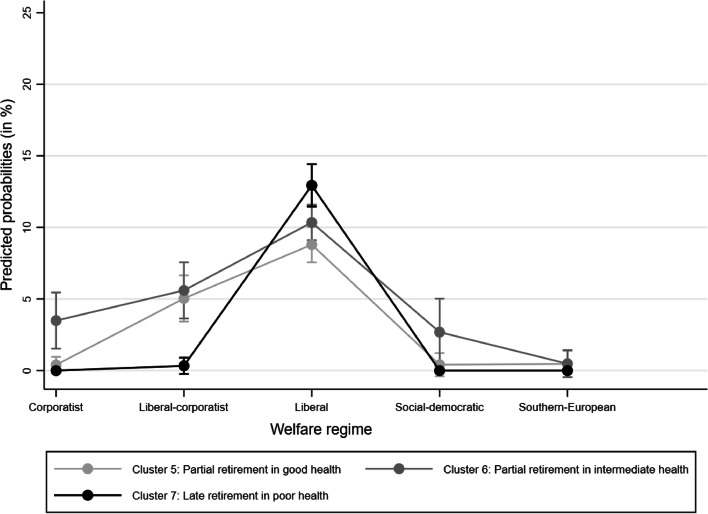
Predicted probabilities (with 95% confidence intervals) for trajectories 5, 6, and 7 by welfare state

With respect to the welfare regime (Fig. 2), our findings revealed two broad patterns. First, Cluster 7 (late retirement in poor health – primarily Chilean) has a very low or zero probability in corporatist, liberal-corporatist, social-democratic, and Southern European welfare regimes. In liberal welfare regimes, however, Cluster 7 shows a probability of 12.5%. These findings not only provide strong evidence for hypothesis H2a, assuming that late retirement in poor health is more likely in liberal welfare regimes than in any other, but also suggest that late retirement in combination with any health status was most likely in liberal welfare regimes. Second, Clusters 5 (partial retirement in good health), 6 (partial retirement in intermediate health), and 7 (late retirement in poor health) were substantially more likely in liberal and, to some extent, liberal-corporatist welfare regimes than in all other regimes. These differences were statistically significant. Within the liberal welfare regime, we observe a high probability for Clusters 5, 6, and 7, and the probabilities do not statistically differ between the Clusters (i.e., the confidence intervals overlap). This result does not explicitly refer to our hypothesis but represents an interesting result with respect to late retirement in the countries examined.

**Fig. 3:**
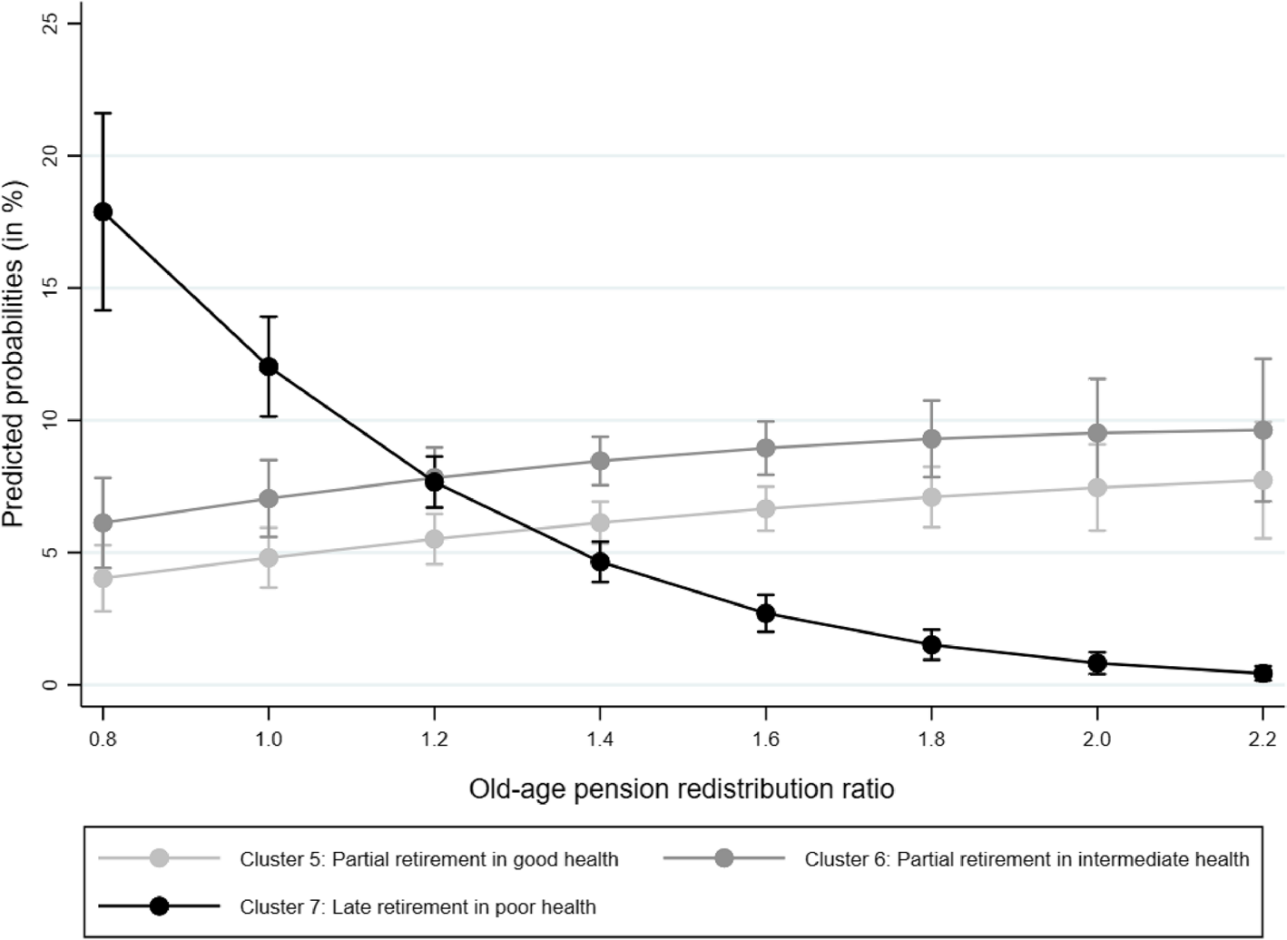
Predicted probabilities (with 95% confidence intervals) for trajectories 5, 6, and 7 by old-age pension redistribution ratio

With respect to old-age pension redistribution (Fig. 3), we find that the level of redistribution is highest in Cluster 5 (partial retirement in good health), second highest in Cluster 6 (partial retirement in intermediate health), and lowest in Cluster 7 (late retirement in poor health – primarily Chilean)). Furthermore, Fig. 3 shows that Cluster 7 is statistically significantly different from Clusters 5 and 6 (the confidence intervals do not overlap). However, Clusters 5 and 6 do not significantly differ from each other at most levels of old-age pension benefit redistribution. At lower levels of old-age pension redistribution, Cluster 7 shows a high probability, and with each increase in the redistribution ratio, Cluster 7 becomes less probable. The opposite pattern can be observed for Clusters 5 and 6. However, the pattern of Cluster 7 versus the patterns of Clusters 5 and 6 are distinct in an important way: the line that draws the distribution of the probabilities of Cluster 7 is much steeper than the lines of Clusters 5 and 6. While low levels of old-age pension redistribution are associated with a very high probability of being in Cluster 7, the probability is more evenly distributed for Clusters 5 and 6. This implies that the risk of being in Cluster 7 is substantial only if there is little old-age pension redistribution. Overall, these findings provide evidence for Hypothesis H2b, which argues that late retirement in poor health is more frequent in countries with comparatively low levels of redistribution. In fact, we found that individuals in a country with a redistribution ratio of 0.8 were four times more likely to be in Cluster 7 than in Clusters 5 or 6. Individuals in a country with a redistribution ratio of 2.2 had a probability of almost zero of being in Cluster 7 and a probability of approximately 10% of being in Clusters 5 or 6.

## Discussion

The increasing labor force participation beyond the SPA in many developed economies has resulted in the need to better understand older people’s late-life employment and health trajectories worldwide. In the current study, relying on the life course perspective and welfare regime theory, we advocated for the investigation of simultaneous employment–health trajectories during the 11-year period after the SPA in 12 OECD countries, and for a better understanding of how welfare regimes and old-age pension redistribution affect these trajectories.

Our results have important theoretical implications for research on the relationship between work and health late in life. First, by modeling individuals’ employment–health trajectories simultaneously over 11 years, we contribute to the literature by stressing the complex interrelationships between the domains of work and health. Our results from the multichannel sequence analysis revealed a large heterogeneity in the combinations of late-life employment and health status. They showed that both early−/on-time retirement as well as late retirement can go along with poor or good health, with around 70% of our sample retiring early/on-time. Based on an analysis of proportions, we have also shown that, contrary to our expectations, late retirement is equally associated with poor health as with good health. This finding nuances earlier research focusing on retirement and health as two distinct life domains, showing that early retirement was associated with poor health [8–10] and bridge employment was positively associated with health [11, 12]. This also extends previous research which models late-life employment and health longitudinally and which does not find that different employment trajectory types are associated with different health developments [21]. In contrast to prior work using conventional sequence analysis and assessing the average level of health within each trajectory type [21], the use of multichannel sequence analysis in our study allowed us to assess heterogeneity in health status within each trajectory type. These heterogeneous effects may be explained by different mechanisms at work at the same time. On one hand, the association between late retirement and good health may indicate that healthy older workers use the possibility to continue working because they are thriving at work [49]. On the other hand, the association between late retirement and poor health may be the result of workers in ill health being forced to continue working, for instance for financial reasons [50].

Second, based on the life course perspective, we extend the literature on comparative research at the international level by providing new evidence on how the association between late-life employment and health is shaped by institutional factors. Overall, we find that, in line with welfare regime theory, the broad institutional context of welfare regimes explains some of the variance in late-life employment–health trajectories. Notably, our finding that in liberal welfare regimes, late retirement trajectories in combination with good and poor health are similarly frequent is in line with a previous study [3] that examined a subset of the countries included in the present study.

Furthermore, we expand the welfare regime literature applied to the retirement context by showing that the retirement-specific measure of old-age pension redistribution better predicts late-life employment–health trajectories. As a concrete example, we find that while late retirement trajectories in combination with all health statuses are most frequent in liberal welfare regimes, the late retirement trajectory in combination with poor health is significantly more likely than in combination with good or intermediate health if the old-age pension redistribution ratios are low (i.e., implying greater inequalities between individuals with higher versus lower pre-retirement earnings). This finding suggests that the welfare regime approach can capture well the duration of working life but less how late-life employment is related to health. Moreover, the analysis of the level of old-age pension fairness provides a novel contribution to the previous literature [3, 21, 43, 51, 52] by showing that when a more retirement-specific policy and a measure of health at the individual level are considered, late retirement, in combination with poor health, is significantly more likely if the old-age pension redistribution ratios are low. This suggests that in countries with low old-age pension redistribution ratios, important shares of older workers with poor health may receive little to no financial support in labor market integration (e.g., part-time disability benefits), thus having to work longer, eventually until they pass away. In contrast, older workers in social-democratic, corporatist, and Southern European welfare regimes, and in countries with higher old-age pension redistribution ratios who decide to retire later, may do so on a more voluntary basis, given that they could benefit from generous pensions if they do not want to work for health reasons, which represents an interesting avenue for future research.

## Limitations

Although the primary strength of our study is that our data were collected via a panel longitudinal design with a sample of 3701 older workers from 12 OECD countries from four representative datasets, our work has at least six limitations. First, the sequence-analytic approach does not allow us to establish causality. Nevertheless, by analyzing employment and health simultaneously – instead of treating health as a simple independent variable –, our study provides evidence on how employment and health are intertwined [13]. Specifically, we show that both early−/on-time retirement as well as late retirement can go along with poor or good health. These different pathways point to diverse causal mechanisms such as poor health leading to early labor force exit or financial constraints to remain in the labor force leading to late retirement in poor health. Second, it was not possible to have an equivalent representation of each welfare regime, since there is large variability in the prevalence of employment beyond SPA between regimes (which is typically higher in the liberal regimes). However, such differences are comparable to those appearing in previous cross-national research focusing on later life [53]. Third, multiple imputations by chained equations to replace missing values in employment and health status were used, which may result in a lower degree of precision as compared to using only observations without missing values. Nevertheless, this approach provides the advantage that observed values in the incomplete cases are retained to make full use of them in the analysis which likely offsets the loss in precision [54]; and we used a rather conservative approach, allowing imputations only if there is maximum of one missing value across the entire period of observation. Fourth, not all clusters show the same degree of country- or gender-variation. In particular, Cluster 7 consists mainly of people living in Chile and a few living in Switzerland (i.e., liberal and liberal-corporatist welfare regimes; see Table A.2). Country-variation would probably have been larger if we would have opted for a cluster solution with a lower number of clusters (e.g., a 5-cluster solution). However, the analysis of the PBC, HG, ASW and HC indexes led to the conclusion that the 5-cluster solution was less robust than the 8-cluster solution (see Fig. A.1). Nevertheless, in the interpretation of the results, it should be kept in mind that Cluster 7 (late retirement in poor health) mainly represents individuals living in Chile. Similarly, Cluster 8 (out of the labor force in heterogeneous health) mainly consists of women. 5th, we used a self-reported measure of health, which may be biased, for instance, by cognitive dissonance [55]. Yet, self-reported health has been shown to be a valid measure of objective health status [56]. 6th, as discussed by Lynch [23], with the methods used in our study we were not able to test cohort differences in employment-health trajectories, but rather to explore life-course patterns within a single cohort. Further research should consider extending our analyses to younger cohorts. Another direction for future research is to examine how detailed aspects of the health care system (e.g., the private-public mix of health care provision or access regulation [57]) are related to late life employment-health trajectories.

## Conclusion

As our study has shown, the transition from employment to retirement in diverse health statuses is embedded in institutional contexts that shape older workers’ individual circumstances in later life [41]. For policymakers from countries with a liberal welfare regime and countries with low old-age pension redistribution ratios, this suggests that they need to be aware that such restrictive retirement pensions are likely to force some parts of older workers to extend their working lives in poor health. This may have important consequences in terms of increasing public health costs.

## Supplementary Information

**Additional file 1: Table A.1.** Overview of the four datasets: **Table A.2.** Distribution of the control variables in the eight clusters of interlocked employment and health trajectories (%), **Table A.3.** Proportions and 95% confidence intervals of the eight clusters of interlocked employment and health trajectories (%), **Fig. A.1.** Selection criteria of cluster solutions, **Fig. A.2.** Sequence index plots of 8 clusters of simultaneous employment-health trajectories.

## Data Availability

-Survey of Health Ageing and Retirement in Europe (SHARE): http://www.share-project.org/home0.html -English Longitudinal Study of Ageing (ELSA): https://www.elsa-project.ac.uk -Health and Retirement Survey (HRS): https://hrs.isr.umich.edu/about -Chilean Social Protection Survey (EPS): https://www.previsionsocial.gob.cl/sps/biblioteca/encuesta-de-proteccion-social/
