# Supplementary material for: Health among workers retiring after the state pension age: a longitudinal and comparative study
Source: BMC Geriatr. 2022 Dec 20;22:984. doi: 10.1186/s12877-022-03690-4 (PMC9764581; doi:10.1186/s12877-022-03690-4)
Supplement: Supplementary file 1 — Additional file 1: Table A.1. Overview of the four datasets: Table A.2. Distribution of the control variables in the eight clusters of interlocked employment and health trajectories (%), Table A.3. Proportions and 95% confidence intervals of the eight clusters of interlocked employment and health trajectories (%), Fig. A.1. Selection criteria of cluster solutions, Fig. A.2. Sequence index plots of 8 clusters of simultaneous employment-health trajectories. [file 12877_2022_3690_MOESM1_ESM.docx]

**Appendices**

**Table A.1**. Overview of the four datasets:

| Year | Datasets waves | | | | Age |
| --- | --- | --- | --- | --- | --- |
|  | SHARE | ELSA | HRS | EPS |  |
| 2004 | 1 | 2 | 7 | 2 | ~ SPA |
| 2005 |  |  |  |  |  |
| 2006 | 2 | - | 8 | 3 |  |
| 2007 |  |  |  |  |  |
| 2008 | - | 4 | 9 |  |  |
| 2009 |  |  |  |  |  |
| 2010 | 4 | 5 | 10 | 4 |  |
| 2011 |  |  |  |  |  |
| 2012 | 5 | 6 | 11 | - |  |
| 2013 |  |  |  |  |  |
| 2014 | 6 | 7 | 12 |  | ~ SPA+11 |
| 2015 |  |  |  | 5 |  |

Note: SPA = State Pension Age, SHARE = Survey of Health, Ageing and Retirement in Europe, ELSA = the English Longitudinal Study of Ageing, HRS = Health Retirement Study, and EPS = *Encuesta de Protección Social*. Waves highlighted in grey indicate those that were excluded from the analyses. ELSA’s Wave 3 was excluded from analyses because, by an unknown reason, survey participants in that wave were asked about their employment status but not about the health status of interest for this study (i.e. self-reported health, as explained below).

*Multichannel Sequence Analysis*

In a first step, we used Multichannel Sequence Analysis (MCSA) to estimate simultaneous employment-health trajectories. MCSA, an extension of Sequence Analysis, is a longitudinal statistical method that allows to reconstruct individual chronological pathways concurrently in two or more domains (1). To this purpose, this method examines how similar or different is every pair of individual sequences in two or more domains, the sequential order in which status changes are experienced, as well as the timing in which status changes are experienced. The results of the comparison of individual sequences is a pairwise distance matrix that summarises the “distance” between the individual sequences. Optimal Matching Analysis method was used to estimate the distances, concretely by using constant substitution costs of 2 and constant indel costs of 1 as usually recommended (2). Here it is worth underlining that given that we use constant, and not weighted, substitution and indel costs, the multichannel sequence analyses conducted was not sensitive neither to the kind of employment statuses or health statuses compared, nor to the ordering of the values in the employment status and the health status.

Over the pairwise distance matrix, we performed a hierarchical cluster analysis to group similar individual trajectories into different types. We specifically used the Ward cluster analysis method (3). To identify the most informative and robust number of types of trajectories we use four selection criteria: Average Silhouette Width (ASW), Point Biserial Correlation (PBC), Hubert’s Gamma (HG), and Hubert’s C (HC). While a higher measure in ASW, PBC and HG indexes means a better cluster solution, a lower measure in HC indicates a better solution. Figure A1 shows the results of the four selection criteria of cluster solutions. Around the eight-cluster solution we observe the highest peaks in PBC, HG and ASW, and the lowest peaks in HC, which indicates that this might be considered as one of the more robust solutions to summarize the pathways followed by 3,699 individuals over 11 years from their SPA.

**References**

1. Gauthier J-A, Widmer ED, Bucher P, Notredame C. Multichannel sequence analysis applied to social science data. Sociol Methodol. 2010;40(1):1–38.

2. Macindoe H, Abbott A. Sequence analysis and optimal matching techniques for social science data. In: Hardy M, Bryman A, editors. Handbook of Data Analysis. SAGE; 2004. p. 387–406.

3. Ward JHJ. Hierarchical grouping to optimize an objective function. J Am Stat Assoc. 1963;58(301):236–44.

**Table A.2**. Distribution of the control variables in the eight clusters of interlocked employment and health trajectories (%)

|  | Cluster 1:  Early/on-time retirement in good health | Cluster 2:  Early/on-time retirement in intermediate health | Cluster 3:  Early/on-time retirement in poor health | Cluster 4:  Early death | Cluster 5:  Partial retirement in good health | Cluster 6:  Partial retirement in intermediate health | Cluster 7:  Late retirement in poor health | Cluster 8:  Out of the labor force in heterogeneous health | p-value |
| --- | --- | --- | --- | --- | --- | --- | --- | --- | --- |
| % | 19.94 | 16.97 | 16.13 | 17.29 | 6.21 | 8.43 | 5.70 | 9.32 |  |
| N | 738 | 628 | 597 | 640 | 230 | 312 | 211 | 345 |  |
| Country, n (% row) |  |  |  |  |  |  |  |  | *** |
| United States | 295 (16.0) | 321 (17.4) | 314 (17.0) | 365 (19.8) | 180 (9.8) | 258 (14.0) | - | 114 (6.2) |  |
| Chile | 7 (1.7) | - | - | 91 (21.5) | 2 (0.5) | - | 210 (49.5) | 114 (26.9) |  |
| Austria | 18 (32.7) | 16 (29.1) | 6 (10.9) | 10 (18.2) | - | - | - | 5 (9.1) |  |
| Belgium | 37 (27.8) | 51 (38.4) | 16 (12.0) | 12 (9.0) | - | 3 (2.3) | - | 14 (10.5) |  |
| France | 23 (27.7) | 31 (37.4) | 13 (15.7) | 6 (7.2) | 1 (1.2) | 6 (7.2) | - | 3 (3.6) |  |
| Germany | 19 (22.1) | 22 (25.6) | 22 (25.6) | 18 (20.9) | 1 (1.2) | 3 (3.5) | - | 1 (1.2) |  |
| England | 185 (32.0) | 75 (13.0) | 138 (23.8) | 66 (11.4) | 44 (7.6) | 34 (5.9) | - | 37 (6.4) |  |
| Switzerland | 20 (44.4) | 12 (26.7) | 5 (11.1) | 5 (11.1) | - | 2 (4.4) | 1 (2.2) | - |  |
| Denmark | 31 (50.0) | 8 (12.9) | 8 (12.9) | 12 (19.4) | - | 3 (4.8) | - | - |  |
| Sweden | 66 (49.3) | 22 (16.4) | 20 (14.9) | 23 (17.2) | 1 (0.8) | 2 (1.5) | - | - |  |
| Italy | 25 (15.4) | 54 (33.3) | 38 (23.5) | 20 (12.4) | 1 (0.6) | 1 (0.6) | - | 23 (14.2) |  |
| Spain | 12 (13.5) | 16 (18.0) | 17 (19.1) | 10 (11.2) | - | - | - | 34 (38.2) |  |
| Education, n (% row) |  |  |  |  |  |  |  |  | *** |
| Primary | 160 (10.9) | 232 (15.8) | 283 (19.2) | 326 (22.1) | 36 (2.4) | 82 (5.6) | 134 (9.1) | 220 (14.9) |  |
| Secondary | 291 (21.4) | 253 (18.6) | 206 (15.2) | 215 (15.8) | 88 (6.5) | 145 (10.7) | 61 (4.5) | 100 (7.4) |  |
| Tertiary | 287 (33.1) | 143 (16.5) | 108 (12.5) | 97 (11.2 ) | 106 (12.2) | 85 (9.8) | 16 (1.9) | 25 (2.9) |  |
| Gender, n (% row) |  |  |  |  |  |  |  |  | *** |
| Women | 403 (20.4) | 338 (17.1) | 314 (15.9) | 252 (12.8) | 117 (5.9) | 151 (7.7) | 71 (3.6) | 325 (16.5) |  |
| Men | 335 (19.4) | 290 (16.8) | 283 (16.4) | 386 (22.3) | 113 (6.5) | 161 (9.3) | 140 (8.1) | 20 (1.2) |  |
| Age at baseline, mean (standard deviation) | 64.0 (2.0) | 64.1 (1.9) | 64.2 (1.9) | 64.6 (1.4) | 64.3 (1.8) | 64.5 (1.5) | 63.3 (2.6) | 62.5 (2.5) | *** |
| Household income quintile, mean (standard deviation) | 3.6 (1.3) | 3.1 (1.3) | 2.7 (1.3) | 2.6 (1.4) | 4.0 (1.1) | 3.5 (1.2) | 2.5 (1.4) | 2.6 (1.4) | *** |
| Marital status, n (% row) |  |  |  |  |  |  |  |  | *** |
| Married / Partnered | 574 (21.1) | 479 (17.6) | 427 (15.7) | 432 (15.9) | 176 (6.5) | 224 (8.3) | 153 (5.6) | 251 (9.2) |  |
| Divorced / Separated | 66 (16.5) | 49 (12.3) | 75 (18.8) | 95 (23.8) | 27 (6.8) | 48 (12.0) | 10 (2.5) | 30 (7.5) |  |
| Never married | 33 (18.2) | 28 (15.5) | 27 (14.9) | 32 (17.7) | 6 (3.3) | 11 (6.1) | 28 (15.5) | 16 (8.8) |  |
| Widowed | 65 (16.2) | 72 (17.9) | 68 (16.9) | 79 (19.7) | 21 (5.2) | 29 (7.2) | 20 (5.0) | 48 (11.9) |  |
| Number of chronic diseases at baseline, mean (standard deviation) | 0.9 (0.9) | 1.3 (1.1) | 2.0 (1.3) | 2.1 (1.5) | 0.9 (1.0) | 1.8 (1.1) | 0.6 (0.7) | 1.3 (1.1) | *** |

Note: N=3,699. The p-value indicates the results from one-tailed χ^2^-test. Significance levels: *p<0.1, **p<0.05, *** p<0.01

**Table A.3**. Proportions and 95% confidence intervals of the eight clusters of interlocked employment and health trajectories (%)

|  | Proportion | SE | 95% confidence interval | |
| --- | --- | --- | --- | --- |
| Cluster 1:  Early/on-time retirement in good health | 0.200 | 0.007 | 0.187 | 0.213 |
| Cluster 2:  Early/on-time retirement in intermediate health | 0.170 | 0.006 | 0.158 | 0.182 |
| Cluster 3:  Early/on-time retirement in poor health | 0.161 | 0.006 | 0.150 | 0.174 |
| Cluster 4:  Early death | 0.172 | 0.006 | 0.161 | 0.185 |
| Cluster 5:  Partial retirement in good health | 0.062 | 0.004 | 0.055 | 0.070 |
| Cluster 6:  Partial retirement in intermediate health | 0.084 | 0.005 | 0.076 | 0.094 |
| Cluster 7:  Late retirement in poor health | 0.057 | 0.004 | 0.050 | 0.065 |
| Cluster 8:  Out of the labor force in heterogeneous health | 0.093 | 0.005 | 0.084 | 0.103 |

Note: N=3,699

**Figure A.1***.* Selection criteria of cluster solutions

Note: The Y axis indicates the normalized outcomes (with Z scores) of the selection criteria, the X axis the number of cluster or types is displayed. We present normalized scores given that some of these criteria have an index that ranges from -1 to 1, while others from 0 to 1. While a higher measure in ASW, PBC and HG indexes means a better cluster solution, a lower measure in HC indicates a better solution (Studer, 2013). Around the eight-cluster solution, we observe the highest peaks in PBC, HG and ASW, and the lowest peaks in HC, which indicates that this might be considered as one of the more robust solutions to summarize the trajectories followed by 3,701 individuals over 11 years from their SPA.

**Figure A.2**. Sequence index plots of 8 clusters of simultaneous employment-health trajectories

|  | **Employment trajectory** | **Health trajectory** |
| --- | --- | --- |
|  | **Early and on-time retirement** | |
|  |  |  |
| Cluster 1: Early/on-time retirement in good health (19.9%) | 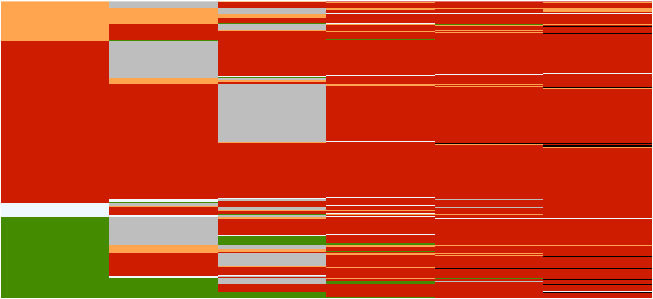 | 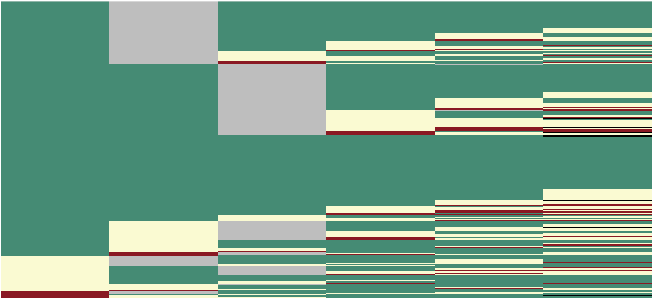 |
| Cluster 2:  Early/on-time retirement in intermediate health (17.0%) | 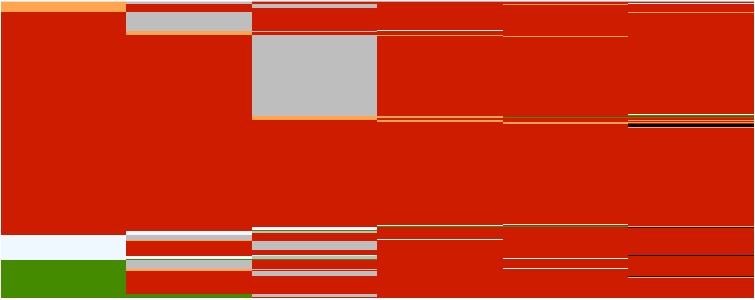 | 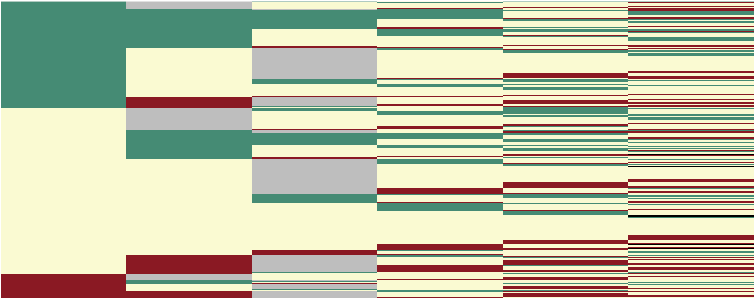 |
| Cluster 3:  Early/on-time retirement in poor health (16.1%) | 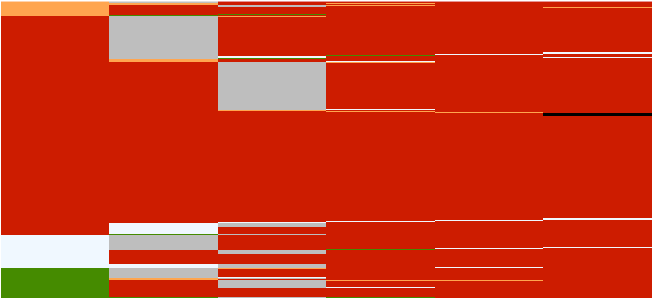 | 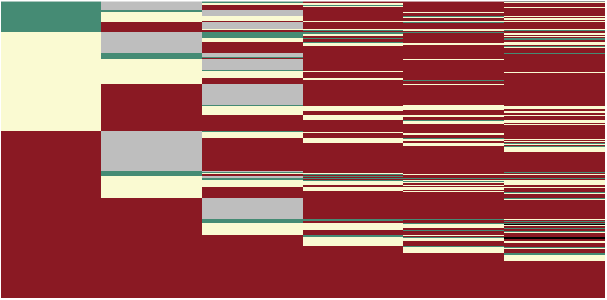 |
| Cluster 4:  Early death (17.3%) | 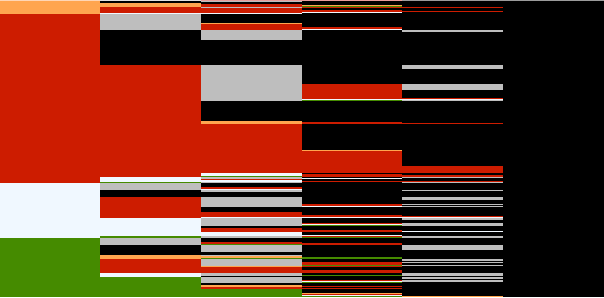 | 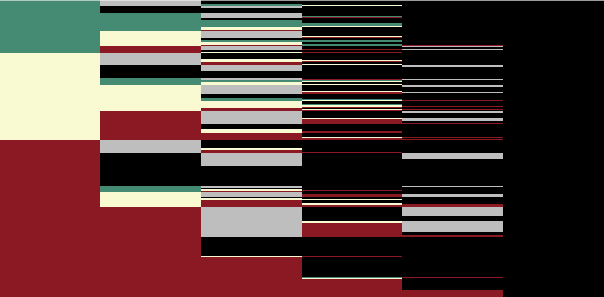 |
|  | **Late retirement** | |
|  |  |  |
| Cluster 5:  Partial retirement in good health (6.2%) | 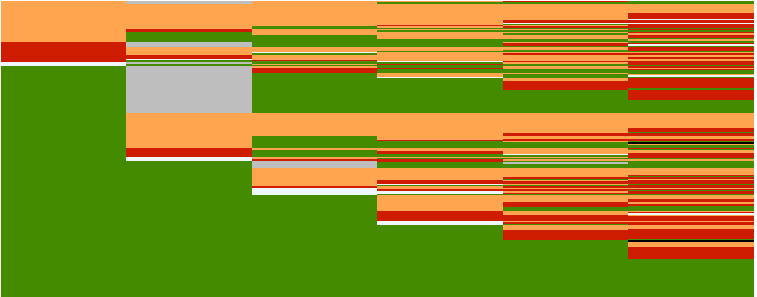 | 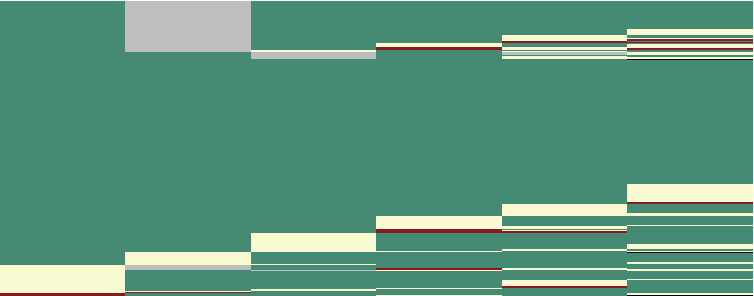 |
| Cluster 6:  Partial retirement in intermediate health (8.4%) | 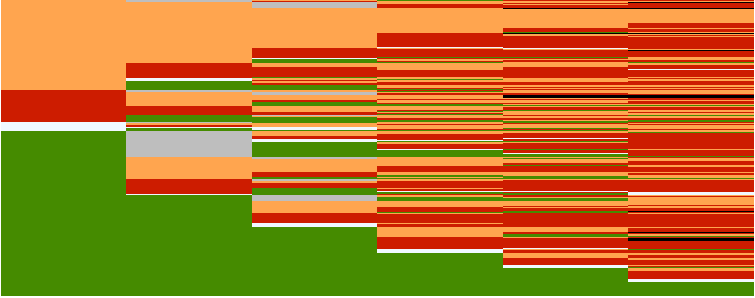 | 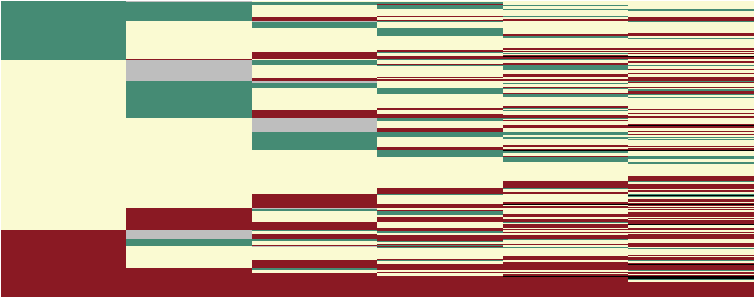 |
| Cluster 7:  Late retirement in poor health (5.7%) | 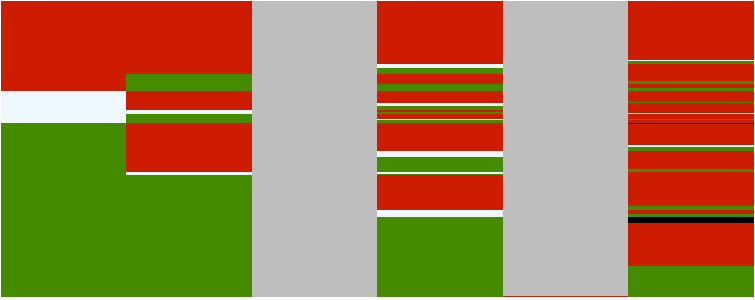 | 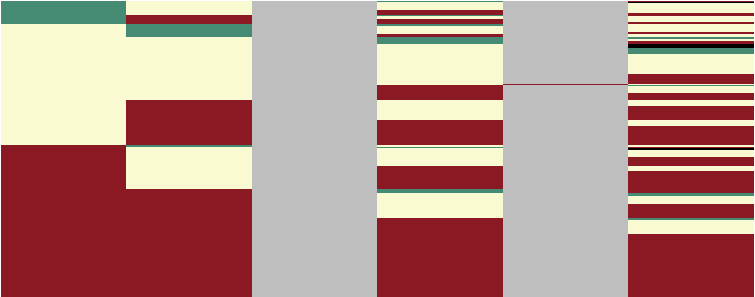 |
|  | **Out of the labor force** | |
|  |  |  |
| Cluster 8:  Out of the labor force in heterogeneous health (9.3%) | 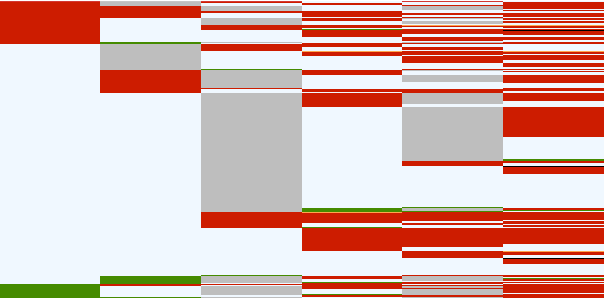  | 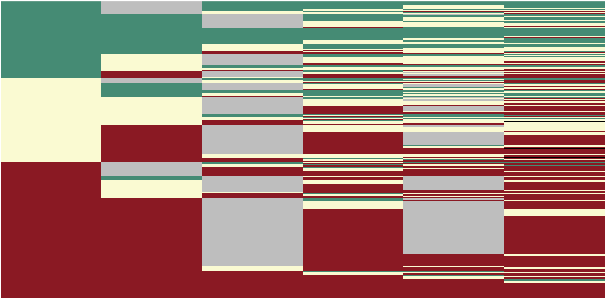 |

Note: ‘Survey gaps’ occur when individuals classified in a trajectory type come from a survey with no waves in a specific year. See Table A.1 to see the waves available in each survey.
